# Body appreciation and resilience are associated with self-esteem among women competing in fitness and bodybuilding: a cross-sectional study

**DOI:** 10.3389/fspor.2026.1948649

**Published:** 2026-08-27

**Authors:** Irene Barbero-Alcocer, Javier González-del-Castillo, Noelia Carbonell-Bernal

**Affiliations:** 1 Department of Education and Teacher Training, Faculty of Law, Education and Humanities, Universidad Europea de Madrid, Villaviciosa de Odón, Madrid, Spain; 2 Department of Sports Sciences, Faculty of Medicine, Health and Sports, Universidad Europea de Madrid, Madrid, Spain

**Keywords:** body appreciation, bodybuilding, cross-sectional study, physique sport, positive body image, resilience, self-esteem, women athletes

## Abstract

**Introduction:**

Women who compete in fitness and bodybuilding participate in a sporting culture where muscularity, leanness, symmetry, and presentation are judged directly. Research has documented eating- and body-related risks, yet much less is known about positive psychological functioning in this population. This study examined how global self-esteem, body appreciation, and perceived capacity to recover from adversity are related, and whether these constructs differ across competitive divisions.

**Methods:**

Item-level questionnaire data were analysed from 107 adult women competing in Bikini Fitness, Bikini Wellness, Bodyfitness/Shape, or Women's Physique/Bodybuilding. Participants completed Spanish versions of the Rosenberg Self-Esteem Scale, Body Appreciation Scale-2, and Brief Resilience Scale. A reproducibility audit identified incorrect reverse scoring in the source self-esteem workbook and omission of one body-appreciation item; all scores were reconstructed from raw responses. Prespecified associations were estimated with Spearman correlations, Holm-adjusted tests, and 10,000-resample bootstrap confidence intervals. Division differences were assessed with Kruskal–Wallis tests. An exploratory multivariable model used HC3 robust standard errors.

**Results:**

Participants were aged 22–56 years (M = 37.33, SD = 7.60). Self-esteem was positively associated with body appreciation (*ρ* = 0.616, 95% CI [0.462, 0.739]) and resilience (*ρ* = 0.559, 95% CI [0.388, 0.701]); body appreciation and resilience were also positively associated (*ρ* = 0.502, 95% CI [0.330, 0.643]; all Holm-adjusted *p* < 0.001). Psychological scores did not differ significantly across divisions. The adjusted model explained 56.4% of observed self-esteem variance (adjusted R^2^ = 0.538). Body appreciation (standardised *β* = 0.598, *p* < 0.001) and resilience (standardised *β* = 0.235, *p* = 0.015) remained independent statistical correlates, whereas age and division did not.

**Discussion:**

A more appreciative relationship with the body and a greater perceived capacity to recover from stress were associated with higher global self-esteem in this specialised sample. The findings counter an exclusively pathologising account of women's physique sport, but they neither exclude coexisting risk nor demonstrate causal protection. Longitudinal studies spanning preparation, competition, and recovery are required to establish temporal direction and practical relevance.

## 1 Introduction

Fitness and bodybuilding are unusual sporting environments because the body is simultaneously the instrument developed through training, the object presented on stage, and the principal basis of competitive judgement. Competitors are evaluated visually on combinations of muscularity, symmetry, proportion, leanness, and presentation rather than by an external performance metric such as time, distance, or points scored. The preparation required to produce a stage-ready physique can involve months of resistance training, dietary restriction, body-mass reduction, posing practice, and tightly managed routines. The visible result is intentionally temporary. It should not be confused with an athlete's habitual body, her health outside the competitive phase, or her psychological experience across a season (1).

The category often called women's physique sport contains several divisions with different aesthetic codes. Bikini divisions usually reward a less muscular presentation than Bodyfitness/Shape or Women's Physique/Bodybuilding, although terminology and judging criteria vary across federations. Division is therefore a meaningful feature of competitive participation, but it is not a direct measurement of muscle mass, body fat, training history, or exposure to evaluation. Body-composition evidence also shows considerable variation within nominal categories and across competitive phases (2). Treating division labels as a simple continuum from psychologically safe to psychologically risky would consequently substitute an administrative classification for the athlete's actual body and experience.

Women negotiate these sport-specific standards within wider cultural ideas about gender and acceptable muscularity. Classic work on the female-athlete paradox described how sporting strength and competence can be valued while women are simultaneously expected to remain recognisably feminine (3). Accounts from women bodybuilders have portrayed muscularity as a source of agency, confidence, and pleasure, but also as an embodied position that can attract scrutiny and require active management of femininity (4). More recent scholarship suggests that muscularity and femininity are not inevitably experienced as contradictions, although the social conditions under which muscular women are accepted remain unequal (5). These tensions make physique sport a particularly useful setting in which to study how women value their bodies and themselves.

Most health research in this population has appropriately focused on risk. Competition preparation can place athletes under physiological and psychological strain, particularly when low energy intake, high training volume, and rapid appearance change converge. In a survey of 158 women physique athletes, gradual dieting was almost universal and food restriction, excessive exercise, and water manipulation were common; more than one third screened at risk of an eating disorder (6). Whitehead et al. (7) likewise documented disordered eating behaviours among women physique athletes. These findings warrant serious attention because competitive practices are embedded in coaching relationships, peer norms, commercial advice, and judging systems rather than being reducible to isolated personal choices.

Preparation and recovery are also temporally organised experiences. Reviews and case evidence describe changes in mood, libido, sleep, social functioning, and preoccupation with food during prolonged preparation, with substantial individual variation (8). Qualitative research indicates that restoration after competition-related weight loss may be experienced as psychologically difficult even when it is physiologically necessary, because the athlete moves away from a body that has been publicly rewarded (9). A measurement taken at one unspecified point in this cycle therefore offers a cross-sectional portrait, not a stable description of the competitor throughout the year.

Risk evidence must not be used to assume that every participant has poor body image or low self-worth. Meta-analytic work across adult sport shows that athletes do not uniformly report greater body-image concern than non-athletes, and differences between sport types are less consistent than is often implied (10). Research focused specifically on highly aesthetic sports nonetheless identifies negative body perceptions as an important correlate of mental-health difficulties for some women athletes (11). Both statements can be true: physique sport may contain salient risks, while many competitors also report pride, competence, social connection, or positive embodiment. A defensible account must examine variation rather than impose either pathology or empowerment as a universal story.

This need for a balanced account has contributed to the growth of positive body-image research. Positive body image is not simply low dissatisfaction and is not equivalent to believing that one's appearance is flawless. It is a multifaceted way of relating to the body that includes acceptance, respect, care, and appreciation of its characteristics and functions (12). A person can experience appearance-related difficulty while retaining respect for the body, and can appreciate the body without approving of every temporary change. This distinction is especially pertinent when an athlete intentionally alternates between developmental, preparation, stage, and recovery physiques.

Body appreciation is one of the best established measurable facets of positive body image. The Body Appreciation Scale-2 (BAS-2) assesses acceptance of, respect for, and favourable orientation towards the body while avoiding a definition based solely on satisfaction (13). Its Spanish adaptation has shown an interpretable unidimensional structure and good psychometric properties (14). Body appreciation is not a clinical diagnosis and the BAS-2 does not divide people into universally valid low, medium, and high categories. Its value in the present context lies in representing gradations in how competitors relate to bodies that are trained, displayed, compared, and altered over time.

Sport can both support and undermine this appreciative relationship. Participation provides opportunities to experience strength, skill, functionality, and belonging; it also exposes the body to comparison, selection, uniform norms, public visibility, and evaluative feedback. Highly trained women from different sports have shown heterogeneous body-image profiles rather than one athlete pattern (15). Student athletes have reported higher state functionality appreciation after practice, illustrating that embodied sport experiences can alter what is salient about the body even over short periods (16). In physique sport, however, functionality and appearance are unusually intertwined: the work the body can do is used to construct the appearance that judges see.

Evidence connecting positive body image to sport-related confidence provides a further rationale for studying body appreciation. Soulliard et al. (17) found that body appreciation and functionality appreciation were related to sport confidence and subjective performance indicators among student athletes. In Jamaican athletes, body appreciation was positively associated with sport confidence and indirectly associated with subjective performance evaluations (18). Those samples were not physique competitors, and sport confidence is not the same construct as global self-esteem. Their findings nevertheless suggest that an appreciative relationship with the body may have relevance beyond appearance satisfaction.

Global self-esteem concerns a person's overall evaluation of worth rather than evaluation of one specific domain. Rosenberg's formulation emphasises self-acceptance and the sense of being worthy, without requiring a belief in superiority (19). Self-esteem is moderately stable yet capable of change, and longitudinal evidence links it to later social, occupational, and health outcomes (20). Its global nature is important here. An athlete may judge that her current conditioning does not meet a competitive standard while maintaining a broader sense of worth; alternatively, repeated public evaluation may allow a narrow body judgement to become disproportionately important to the whole self.

Body appreciation and self-esteem should consequently be related but not treated as interchangeable. General adult evidence associates positive body image with higher self-esteem and other indicators of mental and physical health (21). Exercise participation can contribute to physical self-perceptions and self-esteem, but the pathway depends on how the activity is experienced rather than on exposure alone (22). For women physique athletes, the association may be strengthened because the body is central to sporting identity, or weakened if self-worth is grounded in relationships, work, values, or competence beyond appearance. Direct evidence from this population is limited.

Resilience offers a second perspective on positive psychological functioning. The term is used inconsistently in sport: it can refer to a trait, a capacity, a process of adaptation, or a favourable outcome following adversity. Contemporary reviews argue that sporting resilience is dynamic and develops through interactions between stressors, protective resources, adaptation, and time rather than residing entirely inside the individual athlete (23). A multidisciplinary account likewise locates resilience in changing person–environment systems and warns against inferring it from a single decontextualised score (24).

The Brief Resilience Scale (BRS) adopts a narrower operational definition: the self-reported ability to recover or ‘bounce back’ after stress (25). This perceived recovery capacity is relevant to training setbacks, judges' feedback, injury, demanding preparation, and post-competition transition. It does not reveal which adversities occurred, whether the environment was supportive, how quickly adaptation unfolded, or whether continued exposure was healthy. Throughout this study, resilience therefore refers to the construct captured by the BRS. It is not used as evidence that participants were invulnerable or that they should tolerate preventable harm.

Women athletes' resilience also has a gendered and relational context. A review focused on young women athletes identified personal, interpersonal, and organisational resources that may support adaptation (26). Recent research has described how women can feel compelled to present resilience in systems where resources or recognition are unequal, turning a potentially protective concept into an expectation of silent endurance (27). Physique competitors may similarly draw strength from mastery and community while facing judgement practices or preparation norms that should be changed rather than merely endured.

A positive association between perceived recovery capacity and self-esteem is plausible. People with a stable sense of worth may appraise setbacks as less identity-threatening and recover more readily; successful recovery may in turn reinforce competence and worth. Body appreciation may also be connected to resilience because accepting normal bodily change could reduce the disruption created by movement away from stage condition. The direction of these relationships cannot be resolved in cross-sectional data. Shared causes such as social support, coaching climate, competitive success, self-compassion, or favourable current circumstances are equally plausible.

The three constructs can be organised in a conceptual network without assuming a causal chain. Body appreciation is domain-specific but broad enough to include care and acceptance; self-esteem is a global self-evaluation; and BRS resilience is a judgement about recovery. Each may influence the others over time, and each may be shaped by the sporting environment. For that reason, the present study did not specify a mediation model. Cross-sectional mediation would impose a temporal order not observed in the data and could turn a descriptive covariance pattern into a misleading process claim.

Self-esteem was selected as the outcome in the exploratory regression because the primary substantive question concerned how body- and recovery-related resources covary with whole-person worth. This placement does not grant self-esteem causal priority or make it a clinical endpoint. Reversing the equation and modelling body appreciation would answer a different conditional question with the same cross-sectional limitations. The three bivariate associations therefore remain the principal findings; the regression is a secondary decomposition of shared variation.

Competitive division is often invoked as an explanation for psychological variation, particularly because divisions differ in their visual ideals. Qualitative work with Figure competitors has documented both accomplishment and adverse social experiences (28), while research on weight-management practices suggests that demanding behaviours occur across divisions rather than belonging only to the most muscular categories (6). Division comparisons remain worthwhile as descriptive questions, but they require caution: unequal groups, federation differences, and unmeasured competitive phase can obscure or imitate category effects.

Three evidence gaps guided the present analysis. First, positive psychological constructs have received less attention than eating- and body-related risk in women physique competitors. Second, the relationships among body appreciation, global self-esteem, and perceived recovery capacity have not been quantified clearly in this population. Third, claims about division-specific psychological profiles are not well supported by direct evidence. Addressing these gaps requires a design that preserves continuous information, reports uncertainty, and avoids converting cross-sectional associations into causal effects.

A methodological gap was also evident. Earlier aggregate exports contained scale classifications and results whose scoring could not be assumed correct. Women physique athletes are vulnerable to stereotyped interpretations, so a technical error that creates low self-esteem can have disproportionate substantive consequences. The present work consequently treated item-level verification, transparent correction, and reproducible computation as part of the research contribution rather than as backstage data cleaning.

The primary objective was to examine associations among global self-esteem, body appreciation, and resilience in adult women competing in fitness and bodybuilding. Three hypotheses were specified: body appreciation would be positively associated with self-esteem; resilience would be positively associated with self-esteem; and body appreciation would be positively associated with resilience. Secondary objectives were to describe the three constructs across competitive divisions and to estimate the independent associations of body appreciation and resilience with self-esteem after statistical adjustment for age and division. The secondary analyses were exploratory. No hypothesis concerned digital hate or gender stereotypes because neither exposure was measured in the available questionnaire data.

## 2 Materials and methods

### 2.1 Study design, setting, and reporting framework

This study used a cross-sectional observational design and a reproducible reanalysis of preserved item-level questionnaire records. The analytic unit was the individual competitor. No intervention was delivered, no follow-up observations were available, and no qualitative material was combined with the survey. The design permits estimation of contemporaneous associations and group distributions; it does not permit estimation of change, incidence, temporal sequence, or causal effect.

The manuscript was prepared with reference to the STROBE statement for cross-sectional studies (29). Because the population of interest was women athletes, sex- and gender-related reporting was also considered against the SAGER recommendations (30). The preserved records describe participants as women competitors but do not contain separate variables for sex assigned at birth and gender identity. The term women is therefore used to reflect the recruitment and eligibility language; no sex–gender comparison is possible.

Data were collected in Spain from 1 November to 23 December 2025, after institutional authorisation dated 20 October 2025. The questionnaire was completed online. The September creation date attached to the workbook originated from an older spreadsheet template that was rewritten for this project and was not used to date the observations. The actual collection window was supplied from the study records and confirmed by the lead investigator.

### 2.2 Participants, eligibility, and recruitment

The analytic sample comprised 107 adults described in the source study as women who competed in fitness or bodybuilding. The preserved recruitment description indicates that potential participants were contacted individually through Instagram. This was a purposive convenience strategy intended to reach a specialised and geographically dispersed sporting population for which no complete sampling frame was available.

Eligibility required adulthood and participation in at least one fitness or bodybuilding competition during the previous five years. The retained dataset included age and competitive division but did not record the date of the most recent competition. It was therefore not possible to distinguish competitors currently in preparation, close to a show, in early recovery, or in an extended improvement phase. Federation, amateur or professional status, years of resistance training, years competing, number of competitions, and current injury status were not available consistently.

Competitive division was recorded as Bikini Fitness, Bikini Wellness, Bodyfitness/Shape, or Women's Physique/Bodybuilding. The labels were retained because they represented the categories reported by participants and used in the original study. They were modelled as nominal groups. We did not assign numerical muscularity scores or presume that adjacent labels were separated by equal increments, particularly because judging standards can differ across federations.

All records that met the preserved eligibility description and contained a participant row in the three scale sheets were included. No record was excluded on the basis of the psychological score, and no outlier was removed from the primary analyses. The number of people approached, the number who opened the questionnaire, and the number who declined or started without completing it were not retained. A conventional response rate could consequently not be calculated.

The available dataset fixed the maximum sample size; no prospective power calculation was documented. Rather than applying a *post-hoc* power calculation, which would largely restate the observed p values, the analysis reports confidence intervals around the primary associations and emphasises the limited precision of the smallest division. This justification follows the principle that sample-size adequacy should be evaluated in relation to the inferential aim and information provided (31).

### 2.3 Measures

#### 2.3.1 Demographic and sporting variables

Age in years and self-reported competitive division were available for every participant. Year of birth was retained in the source sheets and used only as part of the record alignment check. It was not included in the analysis in addition to age. No direct indicator of race or ethnicity, education, employment, income, sexual orientation, relationship status, body mass, body composition, menstrual status, or use of performance-enhancing substances was available.

#### 2.3.2 Global self-esteem

The Rosenberg Self-Esteem Scale (RSES) is a 10-item measure of general self-worth and self-acceptance (19). Respondents rated each statement from 1 (strongly disagree) to 4 (strongly agree). Items 2, 5, 8, 9, and 10 are negatively worded and were reverse-scored. Corrected items were summed to yield a total from 10 to 40, with higher values indicating greater global self-esteem. The Spanish version has demonstrated acceptable factor structure and reliability (32).

The RSES was selected because the study question concerns global worth, not confidence limited to sport or satisfaction with appearance. The scale is not a diagnostic instrument. Although descriptive thresholds are sometimes used in applied settings, cut-points are not invariant across populations and were not part of the validated analytic objective. The total was therefore retained as a continuous score.

#### 2.3.3 Body appreciation

The Body Appreciation Scale-2 (BAS-2) contains 10 items assessing acceptance of, respect for, and favourable attitudes towards the body (13). Response options range from 1 (never) to 5 (always). All items are positively keyed; the item mean was calculated when the complete set was present. Possible scores range from 1 to 5, with higher values indicating greater body appreciation. The Spanish translation has shown evidence of unidimensionality, internal consistency, and construct validity (14).

The BAS-2 captures a positive orientation rather than the reverse of body dissatisfaction. It does not establish whether an athlete is satisfied with her current competitive condition, nor does it separately measure appreciation of body functionality. This construct boundary was retained in interpretation: high BAS-2 scores were described as greater body appreciation, not as proof of freedom from eating concerns or appearance pressure.

#### 2.3.4 Resilience

The Brief Resilience Scale (BRS) is a six-item measure of perceived ability to recover from stress (25). Items were rated from 1 (strongly disagree) to 5 (strongly agree). Items 2, 4, and 6 were reverse-scored and the six responses were averaged, giving a possible range from 1 to 5; higher scores represent greater perceived recovery capacity. The Spanish adaptation has shown satisfactory reliability and validity (33).

The BRS operationalises one component of resilience and should not be read as a complete assessment of a dynamic adaptation process. The questionnaire did not establish exposure to a defined adversity, the severity of any stressor, the resources available, or a subsequent adaptation outcome. We therefore use the measure name resilience for brevity while making its recovery-capacity meaning explicit.

Internal consistency was re-estimated in the present sample for every multi-item measure. Cronbach's alpha was reported for continuity with common practice, and McDonald's omega was added because alpha relies on restrictive assumptions that are often overlooked (34). Reliability coefficients describe score consistency in this sample; they do not by themselves demonstrate unidimensionality, validity, or measurement equivalence across competitive divisions. Complete scoring rules and reliability results are reported in Supplementary Table S1.

### 2.4 Data integrity audit and score reconstruction

The original workbook contained separate sheets for RSES, BAS-2, and BRS responses. A structured audit was performed before inferential analysis. Participant rows were aligned across sheets using the original identifier, year of birth, age, and competitive division. All 107 participant rows aligned on those fields. Each analytic record was then assigned a new sequential identifier from 001 to 107 to prevent ambiguous joins.

The audit identified three duplicated source identifiers: 15, 48, and 98 each appeared twice. The paired rows were not duplicate observations: they differed in the accompanying alignment fields and/or item responses. Both rows were retained, and the newly generated sequential identifier was used in all subsequent operations. The analysis did not rely on the source identifier as a unique key.

Two material spreadsheet errors were identified. First, the original RSES formula reversed the five positively worded items instead of the five negatively worded items. This transformed generally favourable response patterns into an erroneously low mean total. Second, the BAS-2 formula summed nine columns and omitted item 10. The source totals and any categories derived from them were therefore considered invalid.

Every RSES and BAS-2 score was reconstructed directly from the raw item responses using the published scoring rules. BRS scores were independently recalculated as an additional verification step. The BRS sheet included formula-only rows below the participant data; these rows lacked identifiers and were excluded. Recalculated totals were range-checked against each scale's theoretical minimum and maximum.

No item responses, age values, or division labels were missing among the 107 aligned records. Because complete item data were available, no imputation, person-mean substitution, or partial-scale scoring rule was required. Values were not winsorised. The corrected continuous scores, not the erroneous totals or low/medium/high labels in earlier exports, formed the single analysis dataset.

The analytical workflow reconstructed each scale, checked row alignment and missingness, estimated every statistic reported in the tables, and used fixed random seeds for bootstrap procedures. This audit trail was retained to ensure consistency from the item-level responses to the reported results. The complete set of data-audit decisions underlying this workflow is reported in Supplementary Table S2.

### 2.5 Statistical analysis

The primary estimands were the three sample Spearman correlations between corrected psychological scores. They describe the direction and strength of monotonic between-person association in the population reached by the study. The division estimands were omnibus differences in score ranks across the four recorded categories. Regression coefficients were conditional mean differences in RSES score under the stated linear model. Defining these quantities prevents correlation, rank comparison, and adjusted mean association from being treated as interchangeable evidence.

Analyses were conducted after the integrity audit and were based on all 107 eligible records. Participant age and corrected scale scores were summarised using the mean, standard deviation, median, interquartile range, observed minimum and maximum, and skewness. Competitive divisions were summarised with counts and percentages. Distributional shape was examined graphically and with Shapiro–Wilk tests; formal normality tests were not used as binary eligibility rules for every method.

Missing-data handling was specified even though the aligned analytic records were complete. Item-level, scale-level, age, and division missingness were counted before scoring. Had incomplete responses been present, they would not have been silently converted to zero or completed by spreadsheet formulas; the pattern and selected scoring rule would have been reported. In the observed data, complete-case and available-case samples were identical at *n* = 107.

Cronbach's alpha and one-factor congeneric McDonald's omega were estimated for the three scales. For omega, items were standardised and the off-diagonal inter-item correlations were fitted under the relation rᵢⱼ = *λ*ᵢλⱼ using bounded nonlinear least squares. Unit-weighted omega was then calculated from the fitted loadings and uniquenesses. The same estimation procedure was applied identically to each scale.

The three directional hypotheses concerned monotonic associations among RSES, BAS-2, and BRS scores. Spearman's rank correlation was selected because the psychological scores were bounded, showed upper-end concentration, and departed from normality. Two-sided p values are reported to avoid claiming prior knowledge of the impossible direction, while interpretation follows the stated positive hypotheses. Percentile 95% confidence intervals were estimated from 10,000 bootstrap resamples with replacement and a fixed seed.

Family-wise error for the three prespecified correlations was controlled with Holm's sequential procedure (35). Both raw and adjusted p values were retained; adjusted values are used for the hypothesis-level conclusion. Correlations between age and each psychological measure were exploratory and were not included in that primary family. Estimates and confidence intervals, rather than thresholds alone, were used to describe magnitude and precision, consistent with recommendations against dichotomous interpretation of statistical significance (36).

Differences among the four nominal competitive divisions were evaluated with Kruskal–Wallis tests for RSES, BAS-2, BRS, and age. Epsilon-squared was reported as an omnibus effect-size estimate, with negative small-sample estimates truncated at zero. Psychological *post-hoc* pairwise tests were contingent on a significant omnibus result and were not performed because none of the three psychological tests met that criterion. Division-specific medians and interquartile ranges are shown to make within- and between-group distributions visible.

An exploratory multiple linear regression used RSES total as the outcome and BAS-2 mean, BRS mean, age, and division as simultaneous predictors. Bikini Fitness was the reference category. The model was intended to estimate conditional associations, not to predict future self-esteem or identify causal determinants. No automated variable selection, interaction search, mediation model, or data-driven categorisation was used.

Unstandardised coefficients, standardised coefficients, and 95% confidence intervals were reported. Heteroscedasticity-consistent HC3 standard errors were used because leverage can affect conventional standard errors in modest samples and HC3 has favourable small-sample properties (37, 38). Standardised coefficients were obtained by scaling each unstandardised slope by the predictor standard deviation and dividing by the outcome standard deviation. The three division indicators were also tested jointly with a robust Wald statistic.

Model diagnostics included variance inflation factors, residual distribution, leverage, externally studentised residuals, Cook's distance, and the Breusch–Pagan test. The primary model retained every observation. A deliberately conservative sensitivity analysis removed cases exceeding the common Cook's-distance screening value of 4/*n* and refitted the same model; this was used to assess stability, not to redefine the sample. Observations were not labelled erroneous solely because they were influential.

All tests were two-sided with *α* = 0.05. Exact p values are provided to three decimal places except for values below 0.001. The verified analytical workflow implemented scoring, reliability estimation, bootstrap correlations, Holm adjustment, ordinary least squares, HC3 covariance estimation, collinearity checks, and influence diagnostics. No result was copied from the erroneous aggregate tables.

### 2.6 Bias and interpretive safeguards

Several bias pathways were considered when specifying the analysis. Convenience recruitment through Instagram can create coverage and self-selection bias because competitors who are visible online or willing to discuss psychological functioning may differ from those not reached. The absence of invitation and non-completion counts prevents quantitative assessment of this selection process. The sample is therefore described as specialised rather than representative.

All psychological constructs were self-reported in the same questionnaire. Shared response style, social desirability, consistency motives, or current mood may inflate associations between measures (39). Confidential administration and subsequent de-identification may reduce some pressures but cannot eliminate common-method variance. The study did not include informant ratings, behavioural indicators, repeated assessments, or a marker variable that could separate construct covariance from method covariance.

The analysis was further protected against overinterpretation by retaining continuous scores, defining the three primary associations before secondary modelling, adjusting their p values as one family, reporting confidence intervals and effect sizes, and labelling regression and division analyses exploratory. Causal verbs such as affects, predicts, protects, or determines were not used for the observed cross-sectional relationships.

### 2.7 Ethical considerations

The Research Ethics Committee of Universidad Europea de Madrid authorised project CI 2025-716, entitled ‘Impact of digital hate and gender stereotypes on the psychological health of women fitness and bodybuilding competitors', on 20 October 2025. Questionnaire data were collected after that decision, from 1 November to 23 December 2025. The present article reports only variables demonstrably available in the survey: age, division, self-esteem, body appreciation, and resilience. It does not claim to measure digital hate, exposure to stereotypes, or their impact.

Before accessing the questionnaire, participants received written information describing the study purpose, the psychological variables assessed, confidential and pseudonymised data handling, the voluntary nature of participation, the right to withdraw without disadvantage, and the options available regarding data retention or deletion following withdrawal. Consent was recorded electronically through a mandatory item asking whether the respondent consented to participate, with Yes, No, and Other response options. All 107 women retained in the analytic sample selected Yes. The file used for manuscript preparation was de-identified and contained no direct personal identifiers; no potentially identifiable data or images are reported. Twelve interviews collected for a separate 2020 academic project were not analysed or quoted, because they belonged to a different sample, time, and ethical context.

## 3 Results

### 3.1 Data integrity and analytic sample

The three questionnaire sheets contained 107 aligned participant records. Every record had complete responses for all 26 psychological items, age, and competitive division; no value was imputed. Three source identifiers were repeated, but the paired rows represented different participants on the accompanying alignment fields and were retained under new sequential identifiers. Formula-only rows without participant identifiers in the BRS sheet were excluded before the analytic file was created.

Reconstruction changed two source scores materially. Correct reversal of RSES items 2, 5, 8, 9, and 10 produced a mean total of 33.20, whereas the incorrect spreadsheet formula had produced 16.80 by reversing the positive items. Recalculation of BAS-2 across all 10 items produced a mean of 4.06; the source total had omitted item 10. All subsequent results use only the reconstructed scores.

Observed reconstructed scores remained within their theoretical ranges. No participant was removed because of an extreme psychological value or influence statistic. The audit therefore corrected computation without narrowing the observed sample.

The complete-case structure meant that each descriptive statistic, correlation, division test, and regression used the same 107 women. Changes between analyses cannot therefore be attributed to shifting subsamples. Earlier percentages based on low, medium, and high labels were not reproduced because their underlying totals were invalid and the cut-points were not part of the present continuous-score analysis.

### 3.2 Participant characteristics

Participants ranged from 22 to 56 years (M = 37.33, SD = 7.60; median = 38, IQR = 31.5–42.0). Bodyfitness/Shape was the largest category (*n* = 34, 31.8%), and Women's Physique/Bodybuilding was the smallest (*n* = 12, 11.2%). The complete distribution is shown in Table 1.

**Table 1:** Participant distribution and age by competitive division.

Competitive division	*n*	%	Age, M (SD)	Age, median (IQR)	Range
Bikini Fitness	30	28.0	34.47 (7.22)	35.0 (30.0–39.8)	22–52
Bikini Wellness	31	29.0	35.23 (7.15)	35.0 (30.5–40.0)	22–48
Bodyfitness/Shape	34	31.8	40.62 (6.50)	39.0 (37.0–45.0)	27–56
Women's Physique/Bodybuilding	12	11.2	40.58 (8.55)	41.0 (36.2–46.2)	23–53

M, mean; SD, standard deviation; IQR, interquartile range. *N* = 107.

Age distributions differed across categories. Bikini Fitness and Bikini Wellness had mean ages of 34.47 and 35.23 years, respectively, compared with 40.62 for Bodyfitness/Shape and 40.58 for Women's Physique/Bodybuilding. This imbalance supported retaining age as an adjustment variable in the exploratory model.

Bikini Fitness contributed 30 participants (28.0%), Bikini Wellness 31 (29.0%), Bodyfitness/Shape 34 (31.8%), and Women's Physique/Bodybuilding 12 (11.2%). The distribution was sufficiently broad to describe all four groups but remained notably unbalanced for inferential comparison.

### 3.3 Score distributions and internal consistency

Corrected self-esteem scores had M = 33.20 (SD = 5.59), median = 35 (IQR = 29–37), and an observed range of 17–40. The distribution was negatively skewed (skewness = −0.84; Shapiro–Wilk W = 0.912, *p* < 0.001), indicating concentration towards higher totals with retained lower-scoring cases.

Body appreciation had M = 4.06 (SD = 0.75), median = 4.30 (IQR = 3.80–4.50), and ranged from 1.50 to 5.00. It showed the strongest upper-end concentration (skewness = −1.42; W = 0.874, *p* < 0.001). Resilience had M = 3.70 (SD = 0.85), median = 3.83 (IQR = 3.17–4.25), and range = 1.33–5.00 (skewness = −0.42; W = 0.967, *p* = 0.010).

Internal consistency was good for RSES (*α* = 0.846, *ω* = 0.859) and BRS (*α* = 0.841, *ω* = 0.843) and excellent for BAS-2 (*α* = 0.937, *ω* = 0.938). Alpha and omega led to the same qualitative judgement for each scale (Table 2). Item-level descriptive statistics are reported in Supplementary Table S3.

**Table 2:** Corrected score distributions and internal consistency.

Measure	Possible range	M (SD)	Median (IQR)	Observed range	Skew	W	*α*	*ω*
RSES total	10–40	33.20 (5.59)	35.00 (29.00–37.00)	17–40	−0.84	0.912	0.846	0.859
BAS-2 mean	1–5	4.06 (0.75)	4.30 (3.80–4.50)	1.50–5.00	−1.42	0.874	0.937	0.938
BRS mean	1–5	3.70 (0.85)	3.83 (3.17–4.25)	1.33–5.00	−0.42	0.967	0.841	0.843

RSES, Rosenberg Self-Esteem Scale; BAS-2, Body Appreciation Scale-2; BRS, Brief Resilience Scale; W, Shapiro–Wilk statistic; α, Cronbach's alpha; ω, one-factor congeneric McDonald's omega. All scores were reconstructed from item responses. *N* = 107.

### 3.4 Prespecified associations

All three prespecified associations were positive and supported after family-wise error correction. Self-esteem was associated with body appreciation, *ρ* = 0.616, bootstrap 95% CI [0.462, 0.739], and with resilience, *ρ* = 0.559, 95% CI [0.388, 0.701]. Body appreciation was also associated with resilience, *ρ* = 0.502, 95% CI [0.330, 0.643]. All Holm-adjusted p values were < 0.001 (maximum adjusted *p* = 3.60 × 10^8^; Table 3). Full scale-level descriptives and associations are reported in Supplementary Table S4.

**Table 3:** Spearman correlations with bootstrap confidence intervals.

Variable pair	*ρ*	Bootstrap 95% CI	Raw *p*	Holm p
Self-esteem — body appreciation	0.616	[0.462, 0.739]	<0.001	<0.001
Self-esteem — resilience	0.559	[0.388, 0.701]	<0.001	<0.001
Body appreciation — resilience	0.502	[0.330, 0.643]	<0.001	<0.001
Self-esteem — age	0.114	[−0.074, 0.300]	0.243	—
Body appreciation — age	0.130	[−0.057, 0.312]	0.181	—
Resilience — age	0.115	[−0.068, 0.294]	0.238	—

Confidence intervals are percentile intervals from 10,000 bootstrap resamples. Holm adjustment was applied to the three prespecified psychological correlations; age correlations were exploratory.

**Figure 1:**
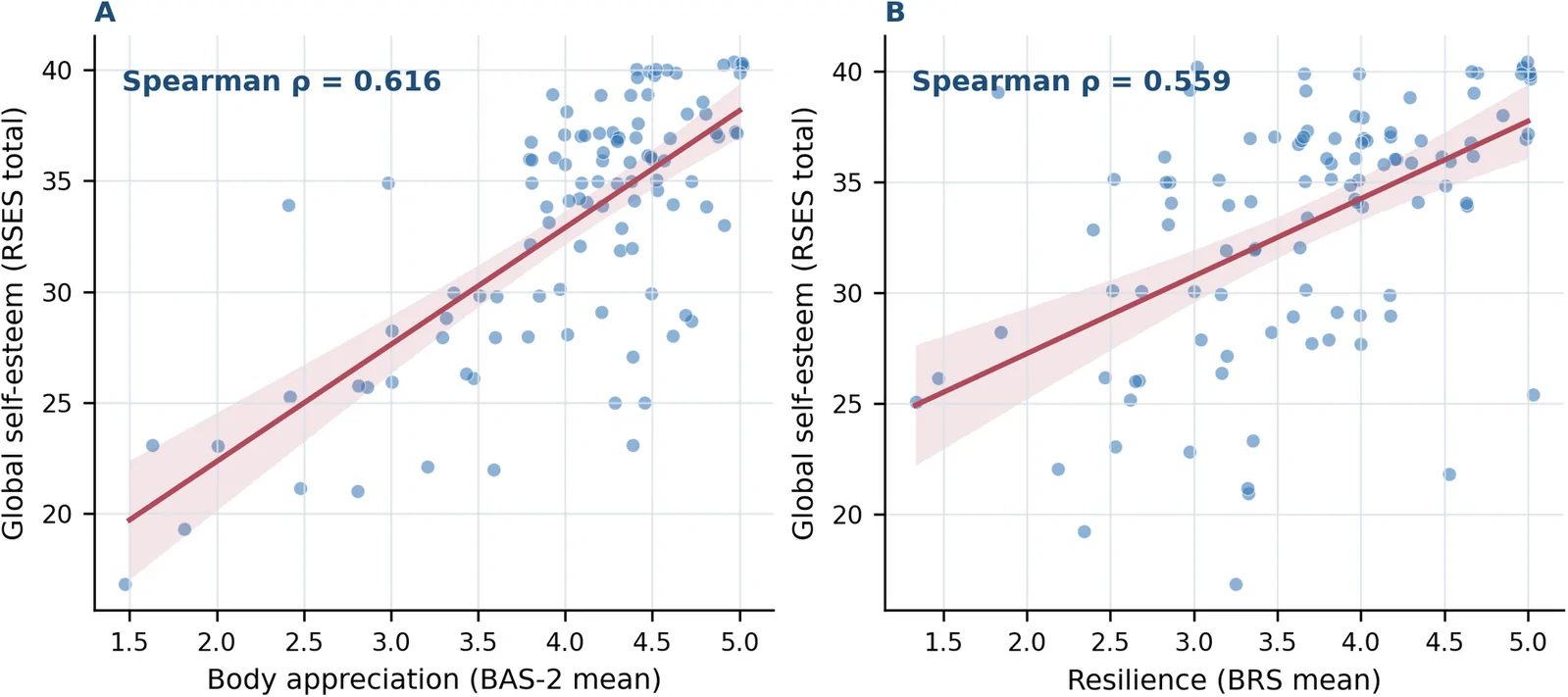
Observed associations of body appreciation and resilience with global self-esteem. **(A)** Body appreciation and global self-esteem. **(B)** Resilience and global self-esteem. Points represent 107 participants and include small deterministic jitter for visibility; all analyses used the unjittered scores. Lines and shaded bands are descriptive ordinary-least-squares trends and 95% confidence bands for the conditional mean. Inferential results in the text and Table 3 are Spearman correlations with bootstrap confidence intervals.

Bootstrap intervals were narrower for the body-appreciation/self-esteem association than would be compatible with a near-zero relationship, but they still allowed meaningful uncertainty in magnitude. The raw data in Figure 1 show both the positive monotonic tendencies and the considerable spread of self-esteem at similar BAS-2 and BRS values. Neither relationship was deterministic.

Age was weakly and non-significantly correlated with self-esteem (*ρ* = 0.114, 95% CI [−0.074, 0.300], *p* = 0.243), body appreciation (*ρ* = 0.130, 95% CI [−0.057, 0.312], *p* = 0.181), and resilience (*ρ* = 0.115, 95% CI [−0.068, 0.294], *p* = 0.238). Each interval crossed zero.

### 3.5 Competitive-division comparisons

Division-specific medians were similar for the three psychological scores (Table 4). RSES medians ranged from 31 in Women's Physique/Bodybuilding to 36 in Bikini Fitness. BAS-2 medians ranged from 4.00 to 4.30, and BRS medians ranged from 3.67 to 4.00.

**Table 4:** Psychological scores and age by competitive division.

Outcome	Bikini Fitness	Bikini Wellness	Bodyfitness/ Shape	Women's Physique/ Bodybuilding	H	*p*	*ε* ^2^
RSES total	36.0 (29.0–37.0)	34.0 (29.5–37.0)	35.0 (32.2–37.0)	31.0 (28.8–36.2)	1.95	0.583	0.000
BAS-2 mean	4.30 (3.82–4.50)	4.30 (3.90–4.50)	4.20 (3.80–4.50)	4.00 (3.80–4.40)	1.14	0.769	0.000
BRS mean	4.00 (3.33–4.46)	3.67 (3.00–4.42)	3.67 (2.88–4.17)	3.83 (3.29–4.00)	1.91	0.591	0.000
Age, years	35.0 (30.0–39.8)	35.0 (30.5–40.0)	39.0 (37.0–45.0)	41.0 (36.2–46.2)	14.21	0.003	0.109

Values by division are median (IQR). H, Kruskal–Wallis statistic with 3 degrees of freedom; ε^2^, epsilon-squared. Negative small-sample ε^2^ estimates were truncated at zero.

Kruskal–Wallis tests did not indicate division differences in self-esteem, H(3) = 1.95, *p* = 0.583, *ε*^2^ = 0.000; body appreciation, H(3) = 1.14, *p* = 0.769, *ε*^2^ = 0.000; or resilience, H(3) = 1.91, *p* = 0.591, *ε*^2^ = 0.000. No psychological pairwise tests were consequently performed.

Age differed among divisions, H(3) = 14.21, *p* = 0.003, *ε*^2^ = 0.109. The age result describes demographic non-equivalence and was not interpreted as a psychological category effect. Category-specific descriptives and the complete omnibus tests are reported in Supplementary Table S5.

### 3.6 Exploratory adjusted model and sensitivity analysis

The full regression was statistically distinguishable from an intercept-only model, F(6, 100) = 21.58, *p* < 0.001. It accounted for 56.4% of observed RSES variance (R^2^ = 0.564; adjusted R^2^ = 0.538). These fit statistics are descriptive of the present sample and model, not estimates of causal explanation.

Body appreciation had the largest adjusted coefficient (B = 4.430, HC3 SE = 0.692, 95% CI [3.074, 5.785], standardised *β* = 0.598, *p* < 0.001). Holding the other model terms constant, a one-unit higher BAS-2 mean corresponded to a 4.43-point higher RSES total.

Resilience retained a smaller independent association (B = 1.548, HC3 SE = 0.638, 95% CI [0.297, 2.799], standardised *β* = 0.235, *p* = 0.015). Age was unrelated to RSES after adjustment (B = 0.001, *p* = 0.993).

None of the three category contrasts with Bikini Fitness was significant, and division was not significant as a joint robust term, Wald *χ*^2^(3) = 3.43, *p* = 0.331. Confidence intervals for the category coefficients were wide, particularly for Women's Physique/Bodybuilding (Table 5).

**Table 5:** Exploratory HC3 regression of global self-esteem.

Term	B	HC3 SE	*β*	95% CI for B	*p*
Intercept	9.599	2.540	—	[4.622, 14.577]	<0.001
Body appreciation	4.430	0.692	0.598	[3.074, 5.785]	<0.001
Resilience	1.548	0.638	0.235	[0.297, 2.799]	0.015
Age, years	0.001	0.055	0.001	[−0.107, 0.108]	0.993
Bikini Wellness	−0.664	1.173	−0.054	[−2.963, 1.634]	0.571
Bodyfitness/Shape	0.694	1.029	0.058	[−1.324, 2.711]	0.500
Women's Physique/Bodybuilding	−1.292	1.660	−0.073	[−4.545, 1.962]	0.436

Outcome: RSES total. Bikini Fitness is the reference division. B, unstandardised coefficient; HC3 SE, heteroscedasticity-consistent standard error; *β*, standardised coefficient; CI, confidence interval. *N* = 107; R^2^ = 0.564; adjusted R^2^ = 0.538. Division joint robust Wald *χ*^2^(3) = 3.43, *p* = 0.331.

Collinearity was low (all variance inflation factors ≤ 1.67). The Breusch–Pagan test did not indicate heteroscedasticity (LM = 3.97, *p* = 0.681). Maximum leverage was 0.150, maximum absolute externally studentised residual was 3.309, and maximum Cook's distance was 0.096. Ten cases exceeded the 4/*n* Cook's-distance screening value, but none approached 1.

Removing those 10 cases for sensitivity purposes yielded *n* = 97 and adjusted R^2^ = 0.727. Body appreciation remained positively associated with RSES (B = 4.845, 95% CI [3.804, 5.885], *p* < 0.001), as did resilience (B = 1.705, 95% CI [0.900, 2.511], *p* < 0.001). Age and division contrasts remained non-significant. The direction and inferential conclusions of the primary model were therefore stable to this influence screen. Full coefficients, diagnostics, and sensitivity results are reported in Supplementary Table S6.

The increase in R^2^ after influence screening was not interpreted as evidence that the reduced model was superior. Removing cases selected because of their relationship to the fitted model necessarily changes fit and can exaggerate apparent regularity. The sensitivity analysis was used only to determine whether the positive BAS-2 and BRS coefficients depended on a small set of influential observations; the all-participant model remains the primary estimate.

## 4 Discussion

### 4.1 Principal findings and contribution

This study provides a corrected and reproducible account of global self-esteem, body appreciation, and resilience in 107 women competing in fitness and bodybuilding. All three prespecified associations were supported after Holm adjustment. Competitors who reported a more appreciative relationship with their bodies tended to report higher global self-esteem; those who reported greater perceived recovery capacity also tended to report higher self-esteem; and body appreciation was positively related to resilience. The confidence intervals excluded zero and placed the three estimates in a moderate-to-large positive range for this sample.

Body appreciation remained the strongest statistical correlate of self-esteem in the exploratory adjusted model. Resilience retained a smaller independent association when body appreciation, age, and division were held constant. Age contributed almost no adjusted association, and the three division indicators were not jointly significant. The model described more than half of observed self-esteem variance, but that figure must not be read as a proportion causally explained: shared method, overlapping self-evaluation processes, omitted variables, and the cross-sectional design all contribute to model fit.

The descriptive correction is itself an important scientific result. The original spreadsheet reversed the wrong RSES items and produced a mean of 16.80, a value that would have supported a narrative of widespread low self-esteem. Correct scoring produced a mean of 33.20. The original BAS-2 formula also omitted its tenth item; the reconstructed mean was 4.06. These are not cosmetic differences. They change the substantive portrayal of the participants from a predominantly impaired group to a sample with generally favourable scores and meaningful between-person variation.

The findings add a positive-functioning perspective to a literature often dominated by risk. This does not invalidate evidence concerning low energy availability, disordered eating, mood disturbance, or difficult recovery. It shows that risk-focused evidence cannot be used to infer uniformly low self-worth or rejection of the body. Positive body image and vulnerability can coexist in the same person, and a cross-sectional scale score cannot settle how that coexistence changes across the competitive cycle.

The pattern is better described as coordinated self-evaluation than as a hierarchy in which one resource mechanically produces another. RSES, BAS-2, and BRS scores all rely on reflective judgements about the self, but they ask about different targets: whole-person worth, the relationship with the body, and recovery after stress. Their correlations indicate related variation without making the measures redundant. The independent BAS-2 and BRS coefficients are consistent with two distinguishable sources of information about self-esteem, although a latent positive self-regard factor or shared response style could also account for part of the pattern.

The results should not be benchmarked against a clinical or population norm that was not sampled. There was no non-athlete comparison group, no women-athlete group from a non-aesthetic sport, and no diagnostic assessment. Statements that the competitors had ‘better’ or ‘worse’ psychological health than other women would therefore exceed the design. The defensible conclusion is internal to the sample: participants varied, and those variations were systematically related.

The upper-end score concentration may have attenuated rather than created some associations by restricting variance, although shared positive wording could work in the opposite direction. RSES includes positively and negatively keyed items, BRS alternates direction, and BAS-2 is wholly positive; the consistent pattern across these formats makes a single acquiescence explanation incomplete. It does not eliminate the broader common-method problem. Multi-method replication is required before the magnitude of the relationships can be considered stable.

### 4.2 Body appreciation in an appearance-evaluated sport

The association between body appreciation and self-esteem is theoretically credible because physique sport makes the body highly salient to identity and evaluation. Unlike simple appearance satisfaction, body appreciation allows respect and care to remain present when the body does not match an ideal at every moment (12). That orientation may help a competitor place stage condition in context: a temporary performance state can be pursued without becoming the only body judged worthy of respect.

The strong association should not be interpreted as evidence that global self-worth is or ought to be body-dependent. Its meaning may differ between athletes. For one competitor, appreciation may reflect gratitude for strength, discipline, health, or bodily capability; for another, favourable answers may be anchored in current appearance. The BAS-2 does not separate these pathways. A future physique-sport study should combine body appreciation with functionality appreciation, contingent self-worth, appearance- and performance-based athletic identity, and qualitative accounts of what competitors understand by respecting their bodies.

Sport research provides some convergent evidence. Positive body image has been associated with confidence and favourable subjective sport experiences in student athletes (17), and body appreciation has been related to sport confidence in Jamaican athletes (18). The present result extends that pattern to global self-esteem in a women-only physique sample. It does not establish that the same mechanism operates because the populations, competitive structures, and outcomes differ.

Contextual body image is particularly relevant. Women athletes can experience their bodies differently in sport and everyday life, and sport-specific evaluation can add information beyond general body-image measures (40). Physique competitors may also distinguish an improvement-season body from a stage body, or an athlete's gaze from a judge's gaze. A single BAS-2 administration averages across those contexts. Repeated state and trait measures would show whether appreciation is stable or moves as body composition and evaluative pressure change.

The high average BAS-2 score is compatible with, but does not prove, an adaptive relationship with the body. Upper-end concentration may reflect genuine acceptance among experienced competitors, self-selection into or continuation within the sport, current phase, or social desirability. People who found the sporting environment incompatible with their well-being may already have left and would not appear in a sample requiring recent competition. This survivor or healthy-participant possibility deserves explicit investigation.

Gendered interpretation is also necessary. Muscular development can challenge narrow expectations of women's bodies while simultaneously being regulated through division-specific standards of presentation. Earlier accounts captured the effort required to negotiate strength and recognisable femininity (3, 4). More recent evidence points to changing possibilities for muscular femininity (5). Body appreciation may partly express agency within these negotiations, but a positive score should not be used to deny the existence of gendered scrutiny.

The result has a further conceptual implication: the stage aesthetic should not be assumed to be psychologically corrosive for every athlete, nor should dedication to it be taken as evidence of positive embodiment. The association observed here concerns differences between people at one time. It cannot reveal whether preparation increased or reduced each woman's appreciation relative to her own baseline. A within-person design could yield a different pattern from the present between-person association.

A distinction between appreciation and appearance-contingent esteem may help explain why physique sport can be experienced as both affirming and precarious. Appreciation provides reasons to value the body that can survive ordinary change; contingent esteem rises and falls with adherence to an ideal or favourable evaluation. Two competitors could obtain similar BAS-2 totals while differing in how conditional that appreciation becomes near a competition. Measuring contingencies explicitly would clarify whether a favourable body relationship buffers stage evaluation or is itself dependent on it.

The social audience around the physique also deserves study. Judges, coaches, training partners, intimate partners, and social-media followers may each reinforce a different body. Praise during extreme leanness can make necessary restoration harder to accept, whereas feedback centred on health, skill, and long-term development may support a broader basis for appreciation. Buechel et al. (9) showed that recovery is not simply the reverse of dieting; it is a psychologically interpreted transition. The present cross-sectional association is compatible with that account but cannot identify which audiences or feedback practices mattered.

Body appreciation may be especially consequential during recovery because the socially rewarded direction of change reverses. During preparation, weight reduction and increasing definition may attract approval; after the show, restoration can be both health-promoting and emotionally difficult. An appreciative stance that includes care could help reconcile those demands, but the present data contain no competition date or repeated assessment. The proposition remains a testable longitudinal hypothesis, not an explanation established by the current coefficient.

A dual-continuum view is preferable to replacing negative body image with a positive score. An athlete may appreciate her body and still experience dissatisfaction, dietary rigidity, or anxiety under particular conditions. Because the study measured BAS-2 but not body dissatisfaction or eating pathology, it cannot locate participants on both continua. Future work should measure them together and test whether appreciation moderates, coexists with, or changes independently from risk.

### 4.3 Resilience: capacity, process, and context

Resilience showed a moderate bivariate relationship with self-esteem and a smaller independent association in the adjusted model. The BRS asks whether respondents feel able to recover after stress. A stable sense of worth could make competitive setbacks less globally threatening, while successful adaptation could reinforce beliefs about competence and personal value. Because both constructs were measured concurrently, reciprocal influence and shared causes remain viable explanations.

The association between body appreciation and resilience suggests that these resources may form part of a broader adaptive profile. Respect for the body could facilitate recovery when appearance changes after competition, while confidence in recovering from difficult experiences could help preserve an appreciative stance. The finding is consistent with neither a simple trait model nor a specific mechanism. No adversity, competitive transition, or recovery trajectory was measured.

Contemporary resilience scholarship cautions against equating a BRS score with the full sporting process. The sporting resilience model places adversity, protective resources, and an adaptive response in an iterative sequence (23). Dynamic perspectives add that resilience can vary across time and levels of the person–environment system (24). The present study measured only a general self-perception at one time, so it cannot establish whether a competitor adapted successfully to a defined challenge.

This distinction matters ethically. Praising women athletes for resilience can inadvertently transfer responsibility from organisations and support systems to the individual. Matheson et al. (27) reported that women may feel forced to be resilient under gendered sporting conditions. In physique sport, unsafe preparation advice, stigmatising feedback, or inadequate recovery support should not be reframed as tests the athlete must simply learn to withstand. The appropriate response to preventable harm is structural improvement, not resilience training alone.

A stronger future model would measure stressor exposure, appraisal, resources, response, and outcome at multiple time points. Candidate resources include autonomy-supportive coaching, social support, financial security, access to qualified nutrition and mental-health care, and the ability to pause or change a preparation plan. Outcomes should include well-being, eating behaviour, menstrual and endocrine health, recovery, sport continuation, and the athlete's own assessment of whether adaptation was desirable.

The relatively smaller adjusted BRS coefficient should not be interpreted as evidence that resilience is less important than body appreciation. Coefficient size depends on scaling, reliability, range, shared variance, and the variables included. BAS-2 and BRS scores were moderately correlated; their simultaneous slopes represent what each contributes after removing linear overlap. They are not a ranking of intervention priorities, and the study did not measure the outcomes to which sporting resilience is most directly relevant.

Resilience may also be phase-specific. The demands of maintaining dietary adherence, receiving an unexpected placing, managing illness, and restoring body mass are qualitatively different. A general BRS score cannot indicate whether an athlete can adapt across all of them. Event-linked assessments could distinguish anticipatory coping, immediate response, and recovery, while qualitative accounts could establish whether the athlete regarded the outcome as growth, compromise, or harm.

The absence of adversity measurement also creates a potential interpretive asymmetry. A high BRS score following severe stress and the same score in a relatively undemanding period do not carry identical information. Likewise, a lower score may reflect temporary depletion rather than a stable personal deficit. Pairing the BRS with event exposure and phase would make comparisons fairer and reduce the risk of attaching a trait label to a situational response.

Nor should resilience be treated as a moral virtue. Recovery speed can be constrained by physiology, resources, cumulative stress, and the severity of what occurred. A lower BRS response does not show weak character, just as a high response does not certify that an environment is safe. This interpretive boundary is important when findings are translated into coach education or athlete screening.

### 4.4 Competitive division and age

Self-esteem, body appreciation, and resilience did not differ significantly across the four divisions. The corresponding epsilon-squared estimates were truncated at zero, and the division indicators were not jointly significant in the adjusted model. At face value, division labels did not separate psychological functioning in this sample. Within-division variation was more prominent than between-division variation.

A null omnibus test does not demonstrate psychological equivalence. The Women's Physique/Bodybuilding group contained only 12 participants, giving limited precision for the category often assumed to experience the greatest muscularity-related scrutiny. Federation was not available, so athletes with different judging standards may have shared a label. Competitive phase and actual body composition were also absent. Each of these limitations can dilute a division effect.

One plausible explanation for the absence of detectable differences is that competitors across divisions participate in a shared physique-sport culture. Although aesthetic targets vary, all four categories involve disciplined resistance training, dietary control, repeated physique monitoring, public appearance evaluation, and exposure to overlapping coaching, peer, federation, and social-media norms. Demanding weight-management practices have been documented across this sport rather than being confined to its most muscular divisions (6). These common experiences may be more psychologically salient than differences in category-specific criteria. Selection and continued participation may also contribute, because women who remain in the sport may share self-regulatory resources or ways of adapting to its demands. These explanations were not tested and should not be interpreted as evidence that category-specific experiences are absent.

The result therefore challenges an overly deterministic use of category. Although divisions encode visual criteria, they do not specify how an athlete interprets those criteria, how long she has competed, the quality of her coaching environment, or the feedback she receives. Qualitative evidence from Figure competitors includes both positive accomplishment and social cost (28). Similar labels can therefore contain different psychological experiences.

Age did differ among divisions, with older mean ages in Bodyfitness/Shape and Women's Physique/Bodybuilding. Age was nevertheless weakly correlated with each psychological score and had an adjusted coefficient effectively equal to zero. This pattern indicates that the division groups were demographically different without showing a simple linear age gradient in self-esteem, body appreciation, or resilience.

Age should not be used as a proxy for experience. A 40-year-old competitor may be in her first season, while a younger participant may have several years of competition. Global self-esteem also follows developmental patterns across adulthood (20), but the 22–56-year range and cross-sectional sample cannot isolate maturation, cohort, or sport selection effects. Direct measures of years training, years competing, competitive level, and phase are necessary.

An adequately powered division analysis would need more than balanced numbers. It should establish that category definitions are comparable, measure rather than assume muscularity and conditioning, and test whether scale items function similarly across groups. It should also consider intersections with age, competitive level, and federation. Without these features, increasing the sample alone could yield a precise estimate of an ill-defined category contrast.

The descriptive table is consequently more informative than a binary statement of no difference. It shows overlapping medians and IQRs while retaining the wider ranges present within categories. Readers can see that the nominal groups did not partition the scores cleanly, yet the small Women's Physique/Bodybuilding group leaves room for uncertainty. Reporting that distribution avoids interpreting *p* > 0.05 as proof of sameness.

### 4.5 Methodological meaning of the scoring audit

The scoring audit deserves attention beyond this dataset. Spreadsheet formulas are often treated as transparent, but a plausible-looking total can conceal item direction errors, omitted columns, copied formulas, or mismatched labels. The incorrect RSES computation reversed the substantive meaning of the sample while still producing values inside a numerically possible range. Range checks alone would therefore not have detected it.

Item-level reconstruction provided several safeguards. Published scoring rules were applied directly to the raw responses, scale scores were regenerated, sheets were aligned using multiple fields, missingness was quantified, and every reported statistic was recalculated from the corrected analytical dataset. Sequential identifiers resolved repeated source labels without deleting valid observations. This process created a traceable chain from item responses to the manuscript tables.

The correction also illustrates how data errors can interact with stereotype. An implausibly high proportion of ‘low self-esteem’ among women bodybuilders might have appeared consistent with a pathologising narrative and therefore escaped scrutiny. Reproducibility is not only a technical matter; it protects participants from interpretations shaped by erroneous expectations. The corrected results should replace all earlier aggregates and derived claims.

Reliability estimates after correction were good to excellent, supporting use of unit-weighted scores in this sample. Reliability does not retroactively validate the original formulas, establish measurement invariance, or prove that each scale has identical meaning for physique competitors and the populations in which Spanish adaptations were validated. These remain separate psychometric questions.

Transparent correction also requires preserving the distinction between source and analytic data. The source workbook was not overwritten. Its formulas, item responses, and repeated identifiers remain available for audit, while the corrected scores were generated separately during analysis. This approach avoids silently replacing historical evidence and permits the discrepancy and its resolution to be independently examined.

### 4.6 Implications for coaching and athlete support

The results suggest that body appreciation and perceived recovery capacity are relevant domains for athlete support, not that they are confirmed treatment targets. Coaches and practitioners can avoid presenting stage condition as a sustainable everyday norm, distinguish performance feedback from personal worth, and recognise that post-competition body change is part of recovery rather than evidence of failure. Language that respects the body's function and health may be preferable to praise contingent only on leanness.

A positive-body-image approach should not become pressure to feel positive at all times. Body appreciation allows difficult feelings and is incompatible with dismissing legitimate concern. Practitioners should make space for ambivalence, especially when an athlete moves away from a publicly rewarded physique. Support is more credible when it acknowledges both commitment to the sport and the costs that some preparation practices can impose.

Resilience support should likewise be relational and structural. The goal is not to make athletes tolerate progressively harsher dieting, criticism, or unsafe advice. It is to provide predictable communication, informed choice, referral pathways, recovery planning, and permission to modify or stop a preparation when health is compromised. A resilient sporting environment distributes responsibility across athlete, coach, federation, and professional support network.

Assessment should span the competitive cycle. A single favourable score can miss deterioration during late preparation or difficulty after a show. Brief repeated assessments of mood, eating concerns, body appreciation, sleep, and perceived recovery could help identify within-person change, provided that screening is confidential and linked to qualified support. The present study did not test such monitoring and cannot establish its effectiveness.

Positive-functioning assessment must complement, not replace, established health surveillance. High self-esteem or resilience does not rule out low energy availability, endocrine disturbance, disordered eating, injury, or substance-related risk. The weight-management practices documented in this sport justify multidisciplinary care where feasible (6). Psychological resources and clinical risk belong in the same conversation without being collapsed into one score.

Sport organisations can also reduce avoidable ambiguity by clarifying judging expectations, educating coaches about health-conscious preparation and recovery, and establishing mechanisms for confidential concern. Division-specific ideals are part of the sport, but feedback need not be humiliating or framed as a judgement of a woman's general worth. These recommendations follow from the wider literature and the associative pattern observed here; they are not intervention outcomes from this study.

Coaches occupy a particularly influential position because they often translate judging criteria into daily behaviour. Training and nutrition guidance can remain performance-focused while avoiding moral language about food, weight, or compliance. Feedback that separates a judging criterion from personal value may help prevent the competition outcome from becoming a global self-evaluation. These practices require coach education and referral boundaries, not an expectation that coaches provide mental-health treatment.

For sport psychologists, the findings support assessment questions rather than a standardised prescription. Useful questions include what the athlete values about her body outside appearance, what changes threaten her sense of worth, how she interprets recovery, and which relationships support or undermine adaptation. Answers may reveal whether high scores represent flexible resources or favourable functioning that is conditional on the current phase.

Implementation should be proportionate to professional competence. Psychological screening requires privacy, referral arrangements, and clear limits on who can access responses. Results should not be used for team selection, judging, or to label an athlete as insufficiently resilient. Support loses its protective value if competitors believe that honest answers can threaten coaching relationships or competitive opportunity.

### 4.7 Strengths

The study addresses an under-researched population and centres positive psychological constructs without denying known health risks. The women-only sample is relevant to the gendered organisation of physique sport and to the scope of Women in Sport. Four competitive divisions were represented, allowing descriptive comparison across a broader range than studies restricted to one category.

The principal methodological strength is the item-level audit. Two substantive scoring errors were located, documented, and corrected rather than hidden in revised totals. All records aligned across scale sheets and contained complete data. Continuous scoring preserved information and avoided unsupported diagnostic categories.

The statistical report prioritises magnitude and uncertainty. Primary correlations include bootstrap confidence intervals and Holm adjustment; division comparisons include effect sizes and group distributions; the regression reports robust standard errors, standardised and unstandardised coefficients, collinearity checks, influence diagnostics, and a sensitivity analysis. All numerical results were generated from the same corrected analytical dataset using a consistent and verified workflow.

Interpretive safeguards are also strengths. Interviews from a different project were excluded, claims about digital hate were removed because the survey did not measure it, and causal language was avoided. The article distinguishes body appreciation from the absence of risk and BRS recovery capacity from resilience as a complete process.

### 4.8 Limitations

The cross-sectional design is the central limitation. All associations were measured at a single time point; therefore, temporal order cannot be established, reciprocal relationships cannot be separated, and the regression does not identify causal effects. Higher body appreciation may support self-esteem, higher self-esteem may support body appreciation, or both may reflect omitted factors. The same problem applies to resilience. Neither the bivariate correlations nor the adjusted regression should be interpreted as demonstrating causation, prediction, mediation, or intervention efficacy.

The purposive Instagram recruitment strategy limits external validity. Competitors without public profiles, those less active online, those who had left the sport, or those unwilling to answer may be underrepresented. The absence of counts for invitations, refusals, and incomplete questionnaires prevents response-rate calculation and comparison of respondents with non-respondents.

The sample size was adequate to estimate the observed primary associations with informative intervals but was modest for category comparison and multivariable adjustment. Only 12 participants were grouped as Women's Physique/Bodybuilding. Null division results are consequently compatible with both small true differences and imprecise estimation. The regression must remain exploratory.

The sample was drawn from a Spanish competitive context, and federation was not recorded. Division names and judging standards cannot be assumed to translate perfectly across organisations or countries. The age distribution and recent-competition criterion may also distinguish these participants from novice lifters, former competitors, or elite professionals.

Competitive phase was not recorded, so participants could not be classified as being in active preparation or competition (in-season), immediate post-competition recovery, or an improvement or off-season phase. This omission is substantial because these periods involve different bodies, behaviours, and psychological pressures. Combining participants assessed at different points in the competitive cycle may have obscured phase-dependent associations or differences between divisions. Current body composition, weight change, energy availability, menstrual status, training load, injury, sleep, and use of performance-enhancing substances were also unavailable. Physiological and psychological state could therefore not be integrated.

Experience and context were measured sparsely. Years of training, years competing, competitive level, number of shows, placing, coaching model, social support, financial pressure, and exposure to criticism were absent. Age and division are weak substitutes for these variables. Omitted-variable bias is likely in the adjusted model.

All constructs were self-reported in one administration. Common-method variance, response consistency, impression management, and current mood may have strengthened correlations. No social-desirability scale, behavioural measure, clinical interview, or repeated assessment was available. The high averages and negative skew for RSES and BAS-2 may also produce ceiling-related loss of sensitivity.

No instrument specifically assessing body dysmorphic disorder or muscle dysmorphia was administered. The study therefore cannot estimate their symptom burden or prevalence, examine their associations with body appreciation, self-esteem, or resilience, or determine whether risk differs across competitive divisions. BAS-2, RSES, and BRS are not diagnostic or disorder-specific screening measures, and favourable scores on these scales do not exclude coexisting dysmorphic symptoms or other forms of psychological vulnerability.

Internal consistency was strong, but this does not establish factorial validity or measurement invariance in women physique athletes. The sample was too small for a stable division-specific invariance analysis, and the Spanish validation samples were not designed around this sporting culture. Future psychometric work should test whether item meaning changes with competitive phase or category.

The preserved dataset used women as an eligibility category but did not separately record sex assigned at birth or gender identity. The study cannot describe gender-diverse participation, compare sex-related physiology, or examine how gender identity shapes experience. This limitation should be addressed in future recruitment and reporting without assuming that sex and gender are interchangeable.

The participant information documented consent to research participation and described confidential, pseudonymised data handling; all 107 women in the analytic sample selected Yes in the mandatory electronic consent item. It did not expressly authorise unrestricted public release of participant-level responses. The de-identified dataset is therefore not openly deposited. This constrains independent reanalysis but avoids extending data use beyond the documented information provided to participants.

Several analytic choices were made after the data already existed. The three primary hypotheses were defined for the reconstructed analysis, not preregistered before collection, and should not be described as confirmatory in the strongest sense. Holm adjustment and transparent labelling reduce but do not remove this limitation. Independent replication with a prospective protocol would provide a stronger test.

The adjusted model assumed additive linear associations and did not test non-linearity or interactions. This restraint avoided an unstable search with 107 observations, but it means that thresholds, diminishing returns, and phase- or division-dependent relationships could not be evaluated. The model also used observed scale scores without accounting for measurement error, so coefficient attenuation and residual covariance are possible.

### 4.9 Future research agenda

The most informative next step is a longitudinal, phase-sensitive cohort. Women should be assessed during an improvement or off-season phase, early and late preparation, immediately around competition, and throughout post-competition recovery. Multilevel models could separate stable between-person differences from within-person change and test whether earlier body appreciation or recovery capacity precedes later self-esteem. Event-linked assessments would also help distinguish anticipatory coping, immediate responses, and subsequent recovery.

Future studies should record federation, division, objective and perceived competitive level, years training, years competing, stage history, and current preparation status. Repeated body-composition and health indicators would allow psychological change to be interpreted alongside the physical cycle. Sampling across clubs, federations, and countries would reduce dependence on social-media visibility.

Measurement should broaden in two directions. Positive functioning can be represented by functionality appreciation, self-compassion, autonomous motivation, sport confidence, social connectedness, and flexible athletic identity. Risk and context can be represented by eating pathology, low energy availability symptoms, mood, contingent self-worth, objectification, body dysmorphic and muscle dysmorphic symptoms, coaching climate, social comparison, harassment, and exposure to digital hostility. Digital hate should be studied with an explicit exposure measure, not inferred from general psychological outcomes.

Body dysmorphic disorder and muscle dysmorphia require explicit, disorder-specific assessment within this framework. Muscle dysmorphia is diagnostically identified as a specifier of body dysmorphic disorder (41). In a small study of 74 female lifters, Hale et al. (42) found that novice and experienced bodybuilders scored higher than recreational fitness lifters on several muscle-dysmorphia dimensions, indicating potential vulnerability but not providing a prevalence estimate for women physique athletes generally. A staged protocol should begin with confidential, culturally and linguistically validated screening measures administered alongside BAS-2, RSES, and BRS at each competitive phase. Continuous symptom scores could then be examined in relation to body appreciation, self-esteem, resilience, division, competitive phase, appearance monitoring, eating pathology, and training or dietary rigidity. Positive screens should not be treated as diagnoses. Estimating prevalence would require confirmation through a structured clinical assessment conducted by a qualified mental-health professional. The protocol should establish referral and immediate-risk procedures in advance, restrict access to appropriate clinical personnel, and ensure that responses cannot be used for selection, judging, or coaching sanctions.

A mixed-method design could explain why women with similar division labels or scale scores have different experiences, but integration must be prospective and ethically coherent. Interviews should come from the same study population, be covered by explicit consent, and be analysed with a transparent qualitative methodology. Historical interviews from another project should not be added merely to make a quantitative article appear more comprehensive.

Intervention research is premature unless the target and mechanism are specified. Body-image programmes in sport provide a useful foundation, but physique competitors require content that acknowledges intentional appearance change, stage judgement, and post-show recovery. A pilot could test a coach-supported programme combining body respect, flexible self-worth, recovery planning, and referral pathways, with adverse events, dysmorphic symptoms, and eating-related outcomes monitored alongside positive measures.

Research should also examine who leaves the sport and why. Cross-sectional samples of active or recently active competitors may omit women whose health, body image, or social experience prompted withdrawal. Including former competitors would help distinguish adaptation from selective retention and would make the long-term meaning of positive scores more credible.

A future protocol should preregister its primary time points, constructs, and estimands, specify how incomplete longitudinal data will be handled, and involve competitors in choosing outcomes that reflect meaningful well-being. Participant involvement is particularly valuable in a field where researchers can otherwise impose assumptions about either pathology or empowerment. Reproducible scoring and a data dictionary should be created before collection rather than reconstructed afterwards.

Comparative sampling would sharpen interpretation. A design including women from non-aesthetic strength sports, women from other judged aesthetic sports, recreational resistance trainers, former physique competitors, and non-athletes could separate effects associated with training, public appearance evaluation, active competition, and selective retention. Such groups should be matched or adjusted on age and relevant background characteristics rather than treated as naturally equivalent.

## 5 Conclusion

Among 107 women fitness and bodybuilding competitors, body appreciation, resilience, and global self-esteem formed a coherent positive associative pattern. Body appreciation showed the strongest relationship with self-esteem and remained independently associated after adjustment for resilience, age, and competitive division. Resilience made a smaller independent contribution. Psychological scores did not differ detectably across the four divisions, although the smallest category was imprecisely represented.

These findings became visible only after an item-level audit corrected reversed RSES scoring and an omitted BAS-2 item. The corrected results reject the earlier portrayal of a sample defined by low self-esteem. They support a more balanced view in which many women competitors report favourable self- and body-related functioning while participating in a sport with documented health and appearance pressures.

The study does not show that body appreciation or resilience causes self-esteem, that high scores eliminate risk, or that competitive divisions are psychologically equivalent. Its practical message is therefore cautious: athlete support should protect body respect and recovery capacity without individualising structural problems or treating stage condition as a year-round norm. Longitudinal research across preparation and recovery is needed to determine whether the observed relationships represent stable differences, phase-dependent change, or reciprocal processes.

The central contribution is disciplined rather than sensational: the study identifies credible associations, corrects a major scoring failure, and defines the boundaries of what the data can support. That combination is essential when research concerns a population frequently described through stereotypes. The competitors' responses support neither an account of universal damage nor one of automatic empowerment; they support careful study of variation, context, and change.

## Data Availability

The datasets presented in this article are not publicly available because the participant information and consent did not authorize unrestricted public release of participant-level responses. Requests to access the data should be directed to the corresponding author.
