# Supplementary material for: Body appreciation and resilience are associated with self-esteem among women competing in fitness and bodybuilding: a cross-sectional study
Source: Front Sports Act Living. 2026 Aug 27;8:1948649. doi: 10.3389/fspor.2026.1948649 (PMC13558144; doi:10.3389/fspor.2026.1948649)
Supplement: Supplementary file 1 [file Table1.docx]

## **Table S1. *Scoring rules and internal consistency***

| Measure | Items | Response format | Reverse-scored items | Composite score | Cronbach’s α | McDonald’s ω |
| --- | --- | --- | --- | --- | --- | --- |
| Rosenberg Self-Esteem Scale (RSES) | 10 | 1 = Strongly disagree to 4 = Strongly agree | Items 2, 5, 8, 9 and 10 | Sum; range 10–40 | 0.846 | 0.859 |
| Body Appreciation Scale-2 (BAS-2) | 10 | 1 = Never to 5 = Always | None | Mean; range 1–5 | 0.937 | 0.938 |
| Brief Resilience Scale (BRS) | 6 | 1 = Strongly disagree to 5 = Strongly agree | Items 2, 4 and 6 | Mean; range 1–5 | 0.841 | 0.84 |

*Note.* Higher scores indicate greater global self-esteem, body appreciation and resilience. All coefficients were re-estimated from the corrected item-level scores (N = 107). The reconstructed RSES total applied reverse scoring to the negatively worded items, and the reconstructed BAS-2 mean included all 10 items.
