# Supplementary material for: Body appreciation and resilience are associated with self-esteem among women competing in fitness and bodybuilding: a cross-sectional study
Source: Front Sports Act Living. 2026 Aug 27;8:1948649. doi: 10.3389/fspor.2026.1948649 (PMC13558144; doi:10.3389/fspor.2026.1948649)
Supplement: Supplementary file 2 [file Table2.docx]

## **Table S2. *Data-audit decisions***

| Audit item | Finding | Decision / correction |
| --- | --- | --- |
| Participant alignment | All 107 rows match across RSES, BAS-2, and BRS files. | Datasets can be merged row-by-row. |
| Original participant IDs | IDs 15, 48, and 98 are duplicated as labels, but rows represent distinct athletes. | Created unique participant_id values 1–107; retained original_id for traceability. |
| Rosenberg Self-Esteem Scale | Previous workbook reversed positive items 1, 3, 4, 6, and 7. | Corrected scoring reverses negative items 2, 5, 8, 9, and 10. |
| BAS-2 | A menudo was confirmed as 4 and Siempre as 5. | Scored as the mean of 10 items (range 1–5). |
| Brief Resilience Scale | Items 2, 4, and 6 require reverse scoring. | Corrected and scored as the mean of 6 items (range 1–5). |
| Sampling | Individual recruitment through Instagram. | Purposive convenience sampling; not random sampling. |
| Eligibility | At least one competition in the preceding five years. | Retained as inclusion criterion. |
| Competitive phase | Variable across athletes and federation calendars. | Treat as an unmeasured source of heterogeneity and limitation. |

*Note.* The unique participant_id variable (001–107) is used for analysis. The original labels are retained only in the restricted audit file and are not included in shared materials.
