# Supplementary material for: Body appreciation and resilience are associated with self-esteem among women competing in fitness and bodybuilding: a cross-sectional study
Source: Front Sports Act Living. 2026 Aug 27;8:1948649. doi: 10.3389/fspor.2026.1948649 (PMC13558144; doi:10.3389/fspor.2026.1948649)
Supplement: Supplementary file 3 [file Table3.docx]

## **Table S3.** *Item-level descriptive statistics*

| Item | n | Mean | SD | Median | Min | Max | Missing |
| --- | --- | --- | --- | --- | --- | --- | --- |
| RSES 1 | 107 | 3.636 | 0.692 | 4.000 | 1 | 4 | 0 |
| RSES 2 | 107 | 1.533 | 0.805 | 1.000 | 1 | 4 | 0 |
| RSES 3 | 107 | 3.607 | 0.626 | 4.000 | 1 | 4 | 0 |
| RSES 4 | 107 | 3.598 | 0.612 | 4.000 | 1 | 4 | 0 |
| RSES 5 | 107 | 1.626 | 0.986 | 1.000 | 1 | 4 | 0 |
| RSES 6 | 107 | 3.206 | 0.877 | 3.000 | 1 | 4 | 0 |
| RSES 7 | 107 | 3.187 | 0.870 | 3.000 | 1 | 4 | 0 |
| RSES 8 | 107 | 2.533 | 1.176 | 2.000 | 1 | 4 | 0 |
| RSES 9 | 107 | 1.869 | 1.010 | 2.000 | 1 | 4 | 0 |
| RSES 10 | 107 | 1.477 | 0.817 | 1.000 | 1 | 4 | 0 |
| BAS2 1 | 107 | 4.168 | 0.906 | 4.000 | 1 | 5 | 0 |
| BAS2 2 | 107 | 3.748 | 0.953 | 4.000 | 1 | 5 | 0 |
| BAS2 3 | 107 | 4.243 | 0.834 | 4.000 | 2 | 5 | 0 |
| BAS2 4 | 107 | 4.056 | 0.909 | 4.000 | 1 | 5 | 0 |
| BAS2 5 | 107 | 4.009 | 1.068 | 4.000 | 1 | 5 | 0 |
| BAS2 6 | 107 | 4.075 | 0.968 | 4.000 | 1 | 5 | 0 |
| BAS2 7 | 107 | 4.075 | 0.939 | 4.000 | 1 | 5 | 0 |
| BAS2 8 | 107 | 4.150 | 0.950 | 4.000 | 1 | 5 | 0 |
| BAS2 9 | 107 | 3.944 | 0.920 | 4.000 | 1 | 5 | 0 |
| BAS2 10 | 107 | 4.103 | 0.990 | 4.000 | 1 | 5 | 0 |
| BRS 1 | 107 | 4.047 | 0.985 | 4.000 | 1 | 5 | 0 |
| BRS 2 | 107 | 2.533 | 1.246 | 2.000 | 1 | 5 | 0 |
| BRS 3 | 107 | 3.626 | 1.170 | 4.000 | 1 | 5 | 0 |
| BRS 4 | 107 | 2.430 | 1.166 | 2.000 | 1 | 5 | 0 |
| BRS 5 | 107 | 3.729 | 1.042 | 4.000 | 1 | 5 | 0 |
| BRS 6 | 107 | 2.252 | 1.190 | 2.000 | 1 | 5 | 0 |

*Note.* Item statistics are reported in the original response direction. Reverse scoring was applied only when computing RSES and BRS composite scores.
