# Supplementary material for: Body appreciation and resilience are associated with self-esteem among women competing in fitness and bodybuilding: a cross-sectional study
Source: Front Sports Act Living. 2026 Aug 27;8:1948649. doi: 10.3389/fspor.2026.1948649 (PMC13558144; doi:10.3389/fspor.2026.1948649)
Supplement: Supplementary file 4 [file Table4.docx]

## **Table S4. *Scale-level descriptive statistics and full Spearman associations***

| Variable | n | Mean | SD | Median | Q1 | Q3 | Min | Max | Skewness |
| --- | --- | --- | --- | --- | --- | --- | --- | --- | --- |
| Age | 107 | 37.327 | 7.601 | 38.000 | 31.500 | 42.000 | 22.000 | 56.000 | 0.025 |
| RSES total | 107 | 33.196 | 5.592 | 35.000 | 29.000 | 37.000 | 17.000 | 40.000 | -0.837 |
| BAS-2 mean | 107 | 4.057 | 0.755 | 4.300 | 3.800 | 4.500 | 1.500 | 5.000 | -1.417 |
| BRS mean | 107 | 3.698 | 0.848 | 3.833 | 3.167 | 4.250 | 1.333 | 5.000 | -0.421 |

*Note.* Q1 and Q3 are the first and third quartiles. No diagnostic cut-offs were used to classify participants.

| Variable 1 | Variable 2 | Spearman ρ | p value | Descriptive interpretation |
| --- | --- | --- | --- | --- |
| RSES total | BAS-2 mean | 0.616 | < 0.001 | Strong positive association |
| RSES total | BRS mean | 0.559 | < 0.001 | Moderate positive association |
| RSES total | Age | 0.114 | 0.243 | Very weak positive association |
| BAS-2 mean | BRS mean | 0.502 | < 0.001 | Moderate positive association |
| BAS-2 mean | Age | 0.130 | 0.181 | Very weak positive association |
| BRS mean | Age | 0.115 | 0.238 | Very weak positive association |

*Note.* All tests were two-sided. Descriptive labels are provided only to aid reading and do not imply universal effect-size thresholds.
