# Supplementary material for: Body appreciation and resilience are associated with self-esteem among women competing in fitness and bodybuilding: a cross-sectional study
Source: Front Sports Act Living. 2026 Aug 27;8:1948649. doi: 10.3389/fspor.2026.1948649 (PMC13558144; doi:10.3389/fspor.2026.1948649)
Supplement: Supplementary file 5 [file Table5.docx]

## **Table S5.** *Category-specific descriptive statistics and Kruskal–Wallis tests*

| Category | Variable | n | Mean | SD | Median | Q1 | Q3 | Min | Max |
| --- | --- | --- | --- | --- | --- | --- | --- | --- | --- |
| Bodyfitness/Shape | Age | 34 | 40.618 | 6.504 | 39.000 | 37.000 | 45.000 | 27.000 | 56.000 |
| Bodyfitness/Shape | RSES total | 34 | 33.941 | 5.416 | 35.000 | 32.250 | 37.000 | 19.000 | 40.000 |
| Bodyfitness/Shape | BAS-2 mean | 34 | 4.065 | 0.801 | 4.200 | 3.800 | 4.500 | 1.800 | 5.000 |
| Bodyfitness/Shape | BRS mean | 34 | 3.632 | 0.925 | 3.667 | 2.875 | 4.167 | 1.833 | 5.000 |
| Women’s Physique/Bodybuilding | Age | 12 | 40.583 | 8.554 | 41.000 | 36.250 | 46.250 | 23.000 | 53.000 |
| Women’s Physique/Bodybuilding | RSES total | 12 | 31.750 | 5.328 | 31.000 | 28.750 | 36.250 | 21.000 | 40.000 |
| Women’s Physique/Bodybuilding | BAS-2 mean | 12 | 3.992 | 0.568 | 4.000 | 3.800 | 4.400 | 2.500 | 4.700 |
| Women’s Physique/Bodybuilding | BRS mean | 12 | 3.708 | 0.396 | 3.833 | 3.292 | 4.000 | 3.167 | 4.167 |
| Bikini Fitness | Age | 30 | 34.467 | 7.219 | 35.000 | 30.000 | 39.750 | 22.000 | 52.000 |
| Bikini Fitness | RSES total | 30 | 33.200 | 6.139 | 36.000 | 29.000 | 37.000 | 17.000 | 40.000 |
| Bikini Fitness | BAS-2 mean | 30 | 3.977 | 0.937 | 4.300 | 3.825 | 4.500 | 1.500 | 5.000 |
| Bikini Fitness | BRS mean | 30 | 3.856 | 0.839 | 4.000 | 3.333 | 4.458 | 1.500 | 5.000 |
| Bikini Wellness | Age | 31 | 35.226 | 7.149 | 35.000 | 30.500 | 40.000 | 22.000 | 48.000 |
| Bikini Wellness | RSES total | 31 | 32.935 | 5.465 | 34.000 | 29.500 | 37.000 | 22.000 | 40.000 |
| Bikini Wellness | BAS-2 mean | 31 | 4.152 | 0.570 | 4.300 | 3.900 | 4.500 | 2.800 | 5.000 |
| Bikini Wellness | BRS mean | 31 | 3.613 | 0.908 | 3.667 | 3.000 | 4.417 | 1.333 | 5.000 |

*Note.* Competitive category was treated as an exploratory administrative grouping variable rather than a validated continuum of muscularity, competitive level, or psychological risk.

| Outcome | Kruskal–Wallis H | df | p value | Epsilon-squared |
| --- | --- | --- | --- | --- |
| RSE total | 1.951 | 3 | 0.583 | 0 |
| BAS-2 mean | 1.136 | 3 | 0.769 | 0 |
| BRS mean | 1.913 | 3 | 0.591 | 0 |
| Age | 14.210 | 3 | 0.003 | 0.109 |

*Note.* The Women's Physique/Bodybuilding subgroup contained 12 participants. Non-significant results should not be interpreted as evidence of equivalence.
