# Supplementary material for: Body appreciation and resilience are associated with self-esteem among women competing in fitness and bodybuilding: a cross-sectional study
Source: Front Sports Act Living. 2026 Aug 27;8:1948649. doi: 10.3389/fspor.2026.1948649 (PMC13558144; doi:10.3389/fspor.2026.1948649)
Supplement: Supplementary file 6 [file Table6.docx]

## **Table S6.** *Exploratory regression model, diagnostics, and influence-screen sensitivity analysis*

| Predictor | B | HC3 SE | z | *p* value | 95% CI lower | 95% CI upper |
| --- | --- | --- | --- | --- | --- | --- |
| const | 9.599 | 2.540 | 3.780 | < 0.001 | 4.622 | 14.577 |
| BAS_score | 4.430 | 0.692 | 6.403 | < 0.001 | 3.074 | 5.785 |
| BRS_score | 1.548 | 0.638 | 2.426 | 0.015 | 0.297 | 2.799 |
| age | 0.001 | 0.055 | 0.009 | 0.993 | -0.107 | 0.108 |
| Bikini Wellness | -0.664 | 1.173 | -0.566 | 0.571 | -2.963 | 1.634 |
| Bodyfitness/Shape | 0.694 | 1.029 | 0.674 | 0.500 | -1.324 | 2.711 |
| Women’s Physique/Bodybuilding | -1.292 | 1.660 | -0.778 | 0.436 | -4.545 | 1.962 |

*Note.* Outcome = RSES total; n = 107; R² = 0.564; adjusted R² = 0.538; robust F = 26.660, p < 0.001. Bikini Fitness was the reference category.

### 1. *Collinearity diagnostics*

| Predictor | VIF |
| --- | --- |
| BAS_score | 1.372 |
| BRS_score | 1.377 |
| age | 1.196 |
| Bikini Wellness | 1.504 |
| Bodyfitness/Shape | 1.665 |
| Women’s Physique/Bodybuilding | 1.328 |

*Note.* VIF values are reported for all non-intercept predictors.

### 2. *Residual and influence diagnostics*

| Diagnostic | Value | *p* value | Interpretation |
| --- | --- | --- | --- |
| Breusch–Pagan LM | 3.970 | 0.681 | Test of heteroscedasticity |
| Breusch–Pagan F | 0.642 | 0.696 | Test of heteroscedasticity |
| Maximum Cook’s distance | 0.096 | — | Threshold 4/n = 0.037 |
| Cases above Cook’s 4/n threshold | 10 | — | Potentially influential cases |
| Maximum leverage | 0.150 | — | Threshold 2p/n = 0.131 |
| Cases above leverage threshold | 5 | — | Potential high-leverage cases |
| Maximum absolute externally studentised residual | 3.309 | — | Values > 3 warrant review |
| Residual mean | < 0.001 | — | Expected approximately zero |
| Residual SD | 3.692 | — | Model residual dispersion |

*Note.* HC3 robust standard errors were used in the primary adjusted model. The externally studentised residual is based on the deleted-case variance; the primary model retained all 107 observations. Diagnostics characterise model assumptions and potentially influential observations.

### 3. *Sensitivity of standard errors*

| Term | B | Conventional SE | HC3 SE | HC3 *p* |
| --- | --- | --- | --- | --- |
| const | 9.599 | 2.685 | 2.540 | < 0.001 |
| BAS_score | 4.430 | 0.573 | 0.692 | < 0.001 |
| BRS_score | 1.548 | 0.511 | 0.638 | 0.015 |
| age | 0.001 | 0.053 | 0.055 | 0.993 |
| Bikini Wellness | -0.664 | 0.993 | 1.173 | 0.571 |
| Bodyfitness/Shape | 0.694 | 1.018 | 1.029 | 0.500 |
| Women’s Physique/Bodybuilding | -1.292 | 1.342 | 1.660 | 0.436 |

*Note.* Coefficient estimates are identical across conventional and HC3 models; the table shows the effect of robust variance estimation on uncertainty.

### 4. *Influence-screen sensitivity analysis*

| Term | B | HC3 SE | 95% CI lower | 95% CI upper | *p* value |
| --- | --- | --- | --- | --- | --- |
| Intercept | 8.325 | 2.056 | 4.296 | 12.354 | < 0.001 |
| BAS-2 mean | 4.845 | 0.531 | 3.804 | 5.885 | < 0.001 |
| BRS mean | 1.705 | 0.411 | 0.900 | 2.511 | < 0.001 |
| Age | −0.018 | 0.045 | −0.107 | 0.072 | 0.699 |
| Bikini Wellness | −0.091 | 0.780 | −1.619 | 1.437 | 0.907 |
| Bodyfitness/Shape | 0.217 | 0.781 | −1.315 | 1.748 | 0.782 |
| Women’s Physique/Bodybuilding | −0.805 | 1.548 | −3.840 | 2.230 | 0.603 |

*Note.* The same model was refitted after removing the 10 cases that exceeded the Cook’s-distance screening value of 4/*n* in the primary model. The sensitivity model included *n* = 97 observations (R² = 0.744; adjusted R² = 0.727). It was used to assess coefficient stability and did not replace the all-participant primary model. Bikini Fitness was the reference category.
